# Epstein-Barr virus-positive inflammatory follicular dendritic cell sarcoma with significant granuloma: case report and literature review

**DOI:** 10.1186/s13000-024-01457-6

**Published:** 2024-02-16

**Authors:** Chenchen Nie, Xun Xie, Hangyan Li, Yangcan Li, Zhihong Chen, Yanchun Li, Zhenfeng Li

**Affiliations:** Department of pathology, the First Affiliated Hospital of Hunan Normal University, Hunan Provincial People’s Hospital, Jiefang West Road, Changsha, 410000 Hunan Province China

**Keywords:** Follicular dendritic cell sarcoma, Epstein-Barr virus-positive inflammatory follicular dendritic cell sarcoma, Inflammatory pseudotumor-like follicular dendritic cell sarcoma, Epithelioid granulomas, Case report

## Abstract

**Background:**

Epstein-Barr virus-positive inflammatory follicular dendritic cell sarcoma (EBV+IFDCS) is a rare disease characterized by mild clinical symptoms and non-specific imaging findings. The diagnosis of the disease depends on pathological diagnosis. However, EBV+IFDCS has a very broad spectrum of histological morphology and immune phenotypes, and its histopathological features have not been fully described by pathologists.

**Case presentation:**

A 59-year-old female, with no significant discomfort, was found to have a splenic mass during a routine physical examination. Microscopic examination at low magnification revealed numerous epithelioid granulomas, amidst which a substantial inflammatory response was observed. Interspersed among the dense inflammatory cells were spindle or oval-shaped cells, distributed sporadically with indistinct boundaries. Under high magnification, these spindle cells had subtle features: smooth and clear nuclear membranes, inconspicuous small nucleoli, and infrequent mitotic figures. Immunophenotypically, the spindle cells expressed CD21 and CD23, and Epstein-Barr encoding region (EBER) in situ hybridization yielded positive results. The inflammatory milieu predominantly consisted of T cells, with a minority of plasma cells expressing IgG4. The confluence of morphological and immunohistochemical findings led to the final pathological diagnosis of EBV+IFDCS in this case.

**Conclusions:**

The presentation of EBV+IFDCS with pronounced granulomatous changes is rare. This morphological variant poses a high risk of misdiagnosis, frequently leading to confusion with other granulomatous diseases. Accurate diagnosis necessitates a comprehensive analysis, integrating immunohistochemistry and in situ hybridization. The case presented here is instrumental in raising awareness and understanding of EBV+IFDCS, with the goal of reducing misdiagnoses and unrecognized cases.

## Introduction

Epstein-Barr virus-positive inflammatory follicular dendritic cell sarcoma (EBV+ IFDCS), also known as inflammatory pseudotumor-like follicular dendritic cell sarcoma (IPT-like FDCS), is a rare, low-grade malignant tumor that is infrequently reported both domestic and international levels. It exhibits a notable predilection for occurrence in women and Asian populations, with a median age of onset at 54.5 years [1] . Unlike the classic FDCS, EBV+ IFDCS commonly manifests in abdominal organs, especially in the liver and spleen. The tumor typically presents with a larger volume, often displaying a gray-white or grayish-yellow color on cut sections, and exhibits relatively clear boundaries from the surrounding tissues. Microscopically, it is characterized by prominent infiltration of lymphoplasmacytic cells, with scattered distribution of tumor cells. The degree of cellular atypia in tumor cells ranges from mild to moderate, with expression of FDC markers and EBER. This disease demonstrates a wide spectrum of variation in both histological morphology and immunohistochemical expression. This article aims to shed light on diagnosing EBV+IFDCS, focusing on a case with significant granulomatous changes. Through a retrospective examination of pathology data and literature review, our goal is to deepen the understanding of EBV+IFDCS and help decrease misdiagnosis and missed diagnoses.

## Case presentation

**Fig. 1:**
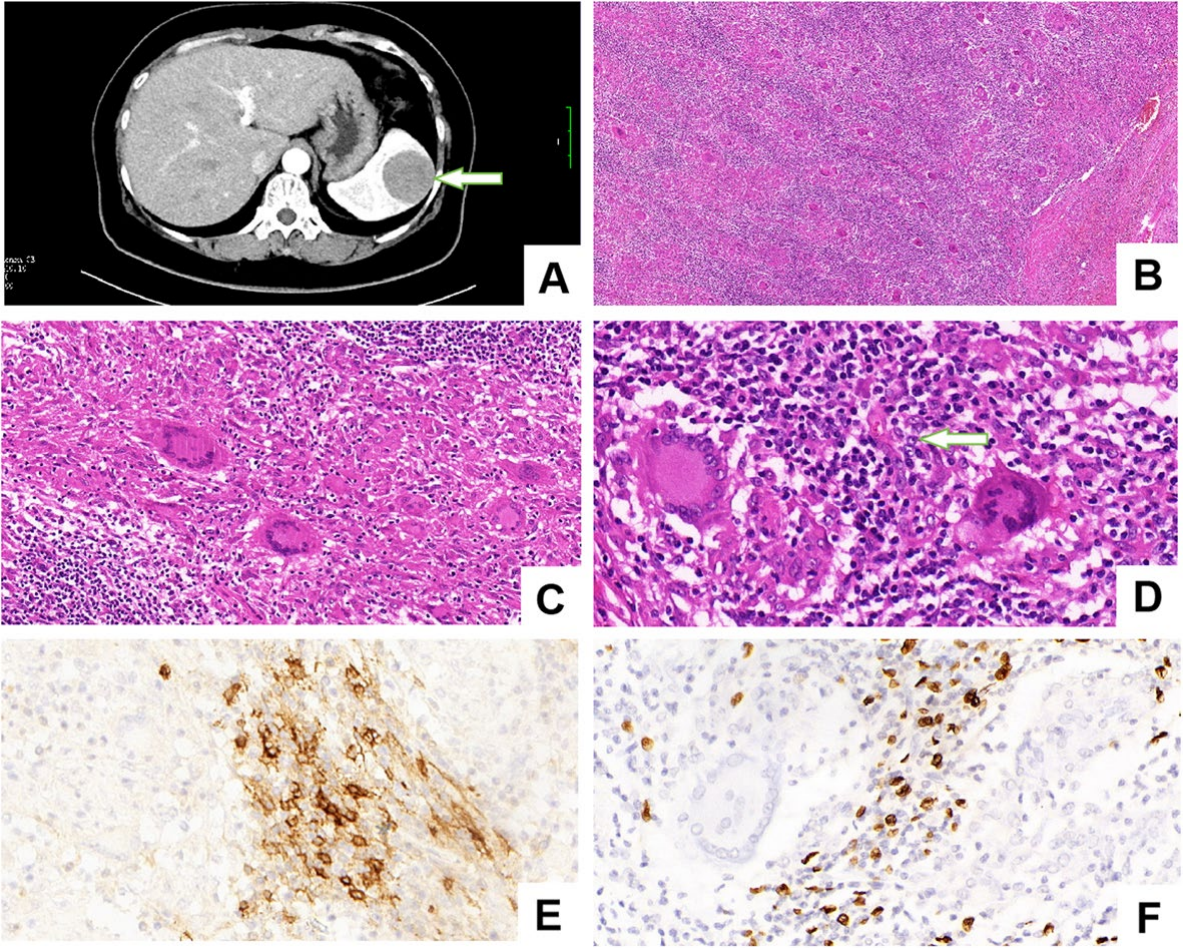
CT scans revealing an iso-low density mass in the spleen (**A**). A large number of diffuse non-caseous epithelioid granulomas in the tumor (**B**. H&E stain, 100x). A large number of diffuse non-caseous epithelioid granulomas in the tumor. HE, Medium magnification (**C**. H&E stain, 200x). Tumor cells are scattered. As indicated by the arrows, the tumor cells display mild cytologic atypia, with nuclei exhibiting a short spindle-shaped or oval morphology. The nuclear membranes are smooth and clear, and the chromatin appears vacuolar with visible small nucleoli. Additionally, mitotic figures are relatively rare (**D**. H&E stain, 400x). The cell membrane of tumor cells exhibits a positive reaction for CD23 (**E**. EnVision method, 400x). EBER positive positivesignals, as revealed by in situ hybridization, under high magnification (**F**. In situ hybridization method, 400x)

This case report involves a 59-year-old female patient who, upon physical examination, exhibited spleen enlargement with occasional left low back pain as the only noticeable symptom. The CT scan indicates a low-density mass in the spleen (Fig. 1A), measuring 41mm in diameter. During the enhancement scan, there is mild and sustained enhancement in each phase, with clear margins. Within the mass, lower-density foci are observable, lacking apparent enhancement. Radiologically, there is a suspicion of sclerosing angiomatoid nodular transformation (SANT) in the spleen. Moreover, no abnormal serum tumor markers were found in this patient. Finally, the patient underwent a partial splenectomy. Macroscopic examination revealed a protruding gray-yellow mass beneath the splenic capsule, measuring 4.5cm×4cm×3cm. The cut surface of the mass exhibited a soft, friable texture, with well-defined boundaries against the surrounding tissues.

Under the microscope, the tumor exhibits relatively clear boundaries with surrounding tissues, lacking an apparent capsule. Within the tumor, there is a widespread distribution of non-caseating necrotizing granulomas (Fig. 1B, C). These granulomas consist of multinucleated giant cells and epithelioid cells, no evident necrosis identified. Interspersed between the granulomatous nodules, there is a significant infiltration of inflammatory cells, predominantly composed of plasma cells and lymphocytes, with the absence of lymphoid follicle formation. In the prominent inflammatory cellular background, spindle or oval-shaped cells are observed. At high magnification, the boundaries between these spindle cells appear indistinct, with a relatively sparse arrangement. The tumor cells exhibit mild cytologic atypia, with chromatin showing a pale affinity for eosin staining. The nuclear shape varies from round to spindle, accompanied by smooth nuclear membranes and conspicuous small nucleoli. Mitotic figures are rarely observed (Fig. 1D).

Immunohistochemical staining further revealed that the spindle cells were positive for CD21 and CD23 (Fig. 1E), while S-100 and SMA demonstrated partial positivity. Desmin, ALK and CKpan, however, were all negative. In the inflammatory stroma, diffuse expanses of CD38-positive plasma cells and CD68-positive tissue cells are evident. Lymphocytes are predominantly composed of CD3-expressing T cells, with localized presence of CD20-positive B cells. Notably, a minimal proportion of plasma cells exhibited positivity for IgG4. The proliferation index of fat fusiform cells, measured by Ki-67, was approximately 15%. Furthermore, Epstein-Barr encoding region (EBER) in situ hybridization confirmed the presence of positive signals for EBV, while acid-fast staining yielded negative results in tumor cells (Fig. 1F).

Combined with morphology and immunohistochemistry, the final pathological diagnosis of this case was EBV+IFDCS. As of the submission of this case, the patient is still alive.

## Discussion

In 1986, researchers led by Monda [2] eported four cases of malignant tumors occurring in lymph nodes, with comprehensive analyses of the tumor cells' morphology, immunohistochemistry, enzyme histochemistry, and ultrastructure consistently indicating an origin from dendritic cells. This marked the first documentation of follicular dendritic cell sarcoma (FDCS). Subsequently, in 2001, scholars including Cheuk [3] proposed classifying FDCS into classic and inflammatory pseudotumor-like types. Classic FDCS predominantly occurs in lymph nodes, is prone to recurrence and metastasis, and has a lower 5-year survival rate. The fifth edition of the World Health Organization classification, however, indicates that classic FDCS primarily occurs in extranodal sites, accounting for about 58%, with lymph nodes being the second most common site at 31% [4]. In contrast, IPT-like FDCS, a distinctive variant with a notable female predominance, presents systemic symptoms, primarily affects intra-abdominal organs such as the liver and spleen, rare occurrences in sites such as the colon, pancreas and lung have also been reported. However, the tumor exhibits an indolent biological behavior, allowing patients to achieve long-term survival even in the case of recurrence. This subtype features dispersed tumor cell distribution, prominent lymphoplasmacytic infiltration, and a close association with Epstein-Barr virus (EBV) infection. Subsequent studies reported additional cases of IPT-like FDCS, progressively deepening our understanding of its broader pathological characteristics. In 2023, the 5th edition of the WHO renamed the inflammatory pseudotumor-like FDCS as EBV-positive follicular dendritic cell sarcoma [4], though the term "sarcoma" remains contentious and has not yet gained widespread acceptance in the pathology community [5].

EBV+ IFDCS is often associated with EBV infection. Research indicates that EBV infection tends to occur before tumor formation, and the immune marker CD21 on FDC is identified as the receptor for EBV, suggesting a pivotal role for EBV in the process of tumor development [3, 6, 7,]. The presence of EBV can also account for the epidemiological characteristics of the disease being more prevalent in East Asia, and the observed deposition of fibrin-like substances in blood vessels reported in numerous cases. Interestingly, in the past decade, cases with EBV-negative IFDCS have also been reported. Beyond EBV infection, IgG4-related diseases and inflammatory pseudotumors are considered associated with the occurrence of EBV+ IFDCS [8], providing an explanation for its tendency in Asian countries and the diversity in immunohistochemical expression. Further research is needed to elucidate the pathogenic mechanisms of this disease.

EBV+ IFDCS often presents with nonspecific clinical manifestations, the patient may have no obvious discomfort or may experience systemic symptoms. The disease progresses slowly, possibly correlated with the tumor's indolent biological behavior. Patients are frequently diagnosed during routine examinations when well-defined masses in the liver or spleen are discovered. Therefore, the authors of this article believe that regular check-ups play a crucial role in the secondary prevention of this disease. This study presents the case of a 59-year-old female who, claiming no apparent discomfort, was incidentally diagnosed with a splenic mass during a routine physical examination, consistent with existing literature findings. Tumors associated with EBV+IFDCS often exhibit painless growth, appearing as single or multiple lesions with well-defined boundaries. Most undergo expansive growth, and may display coagulative necrosis, bleeding, or calcification. Currently, the histopathological characteristics of EBV+ IFDCS have not been thoroughly delineated [9]. Microscopically, it exhibits a wide spectrum of histological morphologies, comprising various histological subtypes, and may also present with features such as granulomas and eosinophilic abscesses [10], Furthermore, there is significant heterogeneity in immunohistochemical expression patterns and staining ranges among different cases. The tumor's histology closely resembles that of inflammatory pseudotumor, characterized by fusiform tumor cells and an abundance of inflammatory cells. These tumor cells, varying in shape from round to oval or spindle, scattered in distribution, and exhibit indistinct boundaries, forming a mat pattern, whirlpool, or bundle arrangement. The cells are enveloped by numerous infiltrating lymphocytes and plasma cells, can organize into lymphoid follicles. In many instances, a significant inflammatory response can obscure tumor cells, potentially leading to confusion with infection or inflammatory pseudotumor. The tumor cells may demonstrate mild to moderate cytologic atypia, with cases presenting binuclear Reed-Sternberg-like cells or tumor giant cells. Nuclei are large, predominantly vacuolar, round or oval, featuring conspicuous nucleoli and smooth nuclear membranes. Mitosis is typically infrequent. Notably, this case presents a unique microscopic morphology characterized by the formation of numerous epithelioid granulomas. This particular feature poses a risk of misdiagnosis as infectious or immune diseases, such as tuberculosis. A literature search revealed 14 cases of EBV+IFDCS with granuloma [10–18] (Table 1), with only 11 cases showing significant granuloma changes [10, 14–18], and a few cases accompanied noticeable tumor necrosis [14, 15, 18]. The reported case in this article did not exhibit obvious necrosis. Further research is needed to explore the reasons for the formation of numerous non-necrotizing granulomas in the tumor. TCases accompanied by a significant formation of granulomas are rare and can be easily misdiagnosed as infectious diseases, emphasizing the importance of careful discrimination by pathologists.

**Table 1:** Clinical data of 14 EBV+IFDCS patients with granuloma reported in previous literature

Case	Gender	Age (y)	Site of involvement	Tumor Size (cm)	Degree of granuloma changes	Reference	First author
1	Female	69	Spleen	6.0	High	[14]	Irena AU
2	Female	49	Spleen	4.7	High	[10]	Li XQ
3	Female	56	Spleen	8.0	High	[10]	Li XQ
4	Female	61	Spleen	8.0	High	[16]	Xue J
5	Female	67	Spleen	5.0	High	[17]	Tian BL
6	-	-	Spleen	-	Low	[12]	Huang YT
7	Male	76	Spleen	3.2	High	[15]	Kim HJ
8	Male	50	Spleen Liver	Spleen: 10.0 Liver: 3.0, 1.5, 1.0	High	[10]	Li XQ
9	Female	42	Liver	2.0, 1.7	High	[10]	Li XQ
10	Female	31	Liver	3.5; 1.7	High	[16]	Xue J
11	Male	38	Liver	8.5	High	[10]	Li XQ
12	Male	38	Liver	12.4	Low	[13]	Zhao S
13	Male	45	Liver	6.7	High	[18]	Wu CY
14	Male	38	Liver	13.5	Low	[11]	Jin K

The tumor expresses at least one characteristic immunohistochemical marker of follicular dendritic cells (FDC), such as CD21, CD23, CD35 and others. In addition, D2-40, SMA, CNA-42 and Clusterin play an auxiliary role in diagnosis and differential diagnosis [19]. However, previous literature indicate numerous cases that do not express FDC markers. Li and other scholars [10] believe that those cases should essentially be reactive myofibroblasts or fibrous tissue cells, rather than neoplastic FDC. They also propose that the minimum diagnostic criteria for EBV+IFDCS should include atypical cells derived from FDC and immunohistochemical markers expressing FDC. It is evident that employing a panel of FDC immune markers is more beneficial for diagnosis than relying on a single marker. Positive EBER in situ hybridization highly suggests the diagnosis of EBV+ IFDCS, Additionally, the identification of Epstein-Barr virus-encoded small RNA (EBER) in situ hybridization positivity can offer supplementary support for a diagnosis strongly indicative of EBV+ IFDCS. Nevertheless, EBER positivity does not constitute an absolute prerequisite for the diagnosis of this condition [20–22].

EBV+ IFDCS requires differentiation from the following diseases: (1) inflammatory pseudotumor : The histological morphology of inflammatory pseudotumor closely resembles that of EBV+IFDCS. However, inflammatory pseudotumor is an inflammatory process, characterized by rare cellular atypia and the absence of expression of follicular dendritic cell (FDC) markers. Immunohistochemistry indicates that most tumor cells are positive for SMA and actin. No FDC markers are expressed, and EBER is negative. (2) Cross-finger dendritic cell sarcoma (Interdigitating dendritic cell sarcoma, IDCS): Although the morphology of the two tumor cells is similar, the inflammatory cell response is less pronounced. S100 is strongly expressed, and FDC markers are not expressed. (3): Both sarcoidosis and this case feature non-caseous granuloma. However, sarcoidosis often involves multiple systems, particularly in the lungs. FDC immunolabeling and EBER are negative in sarcoidosis.

Surgical resection stands as the primary treatment for the disease. Postoperative follow-up, patient reported that she is in good health. The use of radiotherapy and chemotherapy after surgery remains a subject of controversy [23, 24]. Immunotherapy emerges as a promising adjuvant therapy, but its effectiveness requires verification through prospective studies. Due to the tumor's inert biological behavior, the prognosis of the disease is generally favorable, with radical resection significantly improving outcomes.

## Conclusion

In conclusion, EBV+ IFDCS is a rare entity with clinical and radiological presentations exhibiting atypical features. Accurate diagnosis heavily relies on the evaluation of histopathological morphology and immunohistochemical staining. Therefore, a comprehensive understanding of the uncommon and distinctive presentations of EBV+ IFDCS contributes to preventing diagnostic errors or oversights. We report a case of EBV+ IFDCS with pronounced granulomatous changes. Through a review of relevant domestic and international literature, we gathered cases associated with granulomatous changes and analyzed the pathological characteristics of this disease.

## Data Availability

Except for the original data that may reveal the personal information of patients, other data can be obtained from the communication author reasonably.

## Abbreviations

EBV: Epstein-Barr virus
FDCS: Follicular dendritic cell sarcoma
EBV+ IFDCS: Epstein-Barr virus-positive inflammatory follicular dendritic cell sarcoma
IPT: Inflammatory pseudotumor
